# Eye Strain in Health and Disease

**Published:** 1898-10

**Authors:** 


EYE STRAIN IN HEALTH AND DISEASE.—With special Reference to the Cure and Amelioration of Chronic Nervous Derangements without the use of drugs. By Ambrose L. Ramey, A. M., M. D., Author of "Lectures on Nervous Diseases," "The Applied Anatomy of the Nervous System," "Practical Medical Anatomy," etc. Late Professor of the Anatomy of the Nervous System in the New York Post Graduate Medical School and Hospital; Late Professor of Nervous Diseases in the Medical Department of the University of Vermont. Illustrated. Published by the F. A. Davis Co., Philadelphia, New York, and Chicago. 1897. Pages, 321. Price, cloth, $2 net.

The author is the pioneer in the field covered by this book: and many grateful patients, who have been relieved of their sufferings by the application of the truths first advanced by him in his work entitled, Lectures on Nervous Diseases, published in 1888; attest the fact that he is one of the substantial benefactors of his race. He has written many monographs covering his experience in this line during the last ten years, and this present work comprises the substance of them all, and besides is very rich in reported cases. The first chapter discusses the bearing of eye strain on the duration of human life; the second is devoted to the tests of vision and ocular movements; the remaining chapters discuss the relations of eye strain to the treatment of the following conditions: Headache and neuralgia, chorea, insomnia, disturbances of the stomach and digestion, epilepsy, nervous prostration and insanity, etc.

Some of the cases reported show almost wonderful results of the treatment, especially of the epileptic cases. The author thoroughly protests against any medical treatment in epilepsy, until after the "radical elimination of all reflexes" it is found that the fits persist. A tabulation of twenty-six cases of epilepsy treated by this method *alone* shows the following results: Four abandoned treatment and should not be counted. Of the remaing twenty-two cases, seven were completely cured (31.8%), practically cured 3, ameliorated 9, not improved 3 (1.3%).

The work is of great value. J.

